# The PARP1 Inhibitor AZD5305 Impairs Ovarian Adenocarcinoma Progression and Visceral Metastases in Patient-derived Xenografts Alone and in Combination with Carboplatin

**DOI:** 10.1158/2767-9764.CRC-22-0423

**Published:** 2023-03-27

**Authors:** Giulia Dellavedova, Alessandra Decio, Laura Formenti, Mark R. Albertella, Joanne Wilson, Anna D. Staniszewska, Elisabetta Leo, Raffaella Giavazzi, Carmen Ghilardi, Maria Rosa Bani

**Affiliations:** 1 Cancer Metastasis Therapeutics, Oncology Department, Istituto di Ricerche Farmacologiche Mario Negri IRCCS, Milano, Italy.; 2 Bioscience, Oncology R&D, AstraZeneca, Cambridge, United Kingdom.

## Abstract

PARP inhibitors (PARPi) have changed the management of patients with ovarian cancer and their effectiveness has been demonstrated especially in homologous recombination repair–deficient tumors. These first-generation drugs target PARP1, but also PARP2 and other family members potentially responsible for adverse effects that limit their therapeutic potential and restrict their use in combination with chemotherapeutic agents.

We investigated ovarian cancer patient-derived xenografts (OC-PDXs) to assess whether malignant progression could be impaired by a novel inhibitor selective for PARP1 (AZD5305) and to assess the potential of its combination with carboplatin (CPT), the standard-of-care for patients with ovarian cancer.

In *BRCA*-mutated OC-PDXs, AZD5305 achieved greater tumor regressions and longer duration of response as well as a superior impairment of visceral metastasis and improved survival benefit compared with the first-generation dual PARP1/2 inhibitors.

The combination of AZD5305 plus CPT was more efficacious than single agents. Subcutaneously growing tumors experienced regression that persisted after therapy stopped. Combination efficacy was greater against tumors that did not respond well to platinum, even at a dose at which AZD5305 monotherapy was ineffective. The combination therapy impaired metastatic dissemination and significantly prolonged the lifespan of mice bearing OC-PDXs in their abdomen. This combination benefit was evident even when CPT was used at suboptimal doses, and was superior to full-dose platinum treatment.

These preclinical studies demonstrate that the PARP1-selective inhibitor AZD5305 retains and improves the therapeutic benefit of the first-generation PARPi, providing an opportunity to maximize benefits for this class of anticancer agents.

**Significance:**

Selective PARP1i AZD5305 can exceed the efficacy of first-generation PARPi, which target both PARP1 and PARP2, and potentiates the efficacy of CPT when given in combination. AZD5305 alone or in combination with platinum delayed visceral metastasis, ultimately extending the lifespan of OC-PDX–bearing mice. These preclinical models mimic the progression of the disease occurring in patients after debulking surgery, and are translationally relevant.

## Introduction

PARP inhibition has been shown to be synthetically lethal with a deficiency in *BRCA1/2* (1, 2). Accordingly, a number of PARP inhibitors (PARPi) have been developed, showing clinical effectiveness mainly for cancers (i.e., breast, ovarian, pancreatic, prostate) with deleterious mutations in *BRCA* genes, or other deficiency in the homologous recombination repair (HRR) pathway (3, 4). While generally well tolerated, use of PARPi can lead to adverse events in some patients (primarily myelosuppression), leading to dose reductions and limiting their use in combination with other cytotoxic agents.

PARPi (olaparib and niraparib) now offer a major treatment option for women with ovarian cancer, improving outcomes in both the recurrent and frontline settings as maintenance therapy after primary adjuvant therapy with platinum-taxane, and their use has become increasingly widespread (4–6). Despite providing a clinically meaningful progression-free survival (PFS) and overall survival benefit in the first-line setting, there remains an unmet clinical need to further improve patient outcomes. The identification of regimens for patients who appear to benefit less from PARPi is a priority.

To increase the therapeutic utility of PARPi, their combination potential with VEGF inhibition has been tested in clinical trials (7, 8). This has resulted in olaparib plus bevacizumab being approved for maintenance treatment in HRR-deficient tumors (9, 10). Combinations of PARPi with other antineoplastic agents (e.g., platinum, topotecan, temozolomide; refs. 11–15) form an area of great interest because the repair of chemotherapy-induced DNA damage could be weakened when a PARPi is administered simultaneously, leading to improved responses. A phase III trial has shown significantly longer PFS with veliparib added to standard first-line chemotherapy (16). However, due to the frequency and severity of adverse events, to date no PARP1/2i have been approved for use in combination with chemotherapy (17).

All the approved PARPi affect both PARP1 and PARP2, and in some cases other members of the PARP enzyme family (18). Studies suggest that synthetic lethality with *BRCA* mutations is solely driven by inhibition and trapping of PARP1 (19, 20) while PARP2 may in fact be associated with hematologic toxicity (21). Hence, selective targeting of PARP1 while sparing PARP2 might lower the risk of hematologic toxicity and improve the therapeutic index, potentially expanding combination options.

AZD5305 is a new generation, highly potent PARP1 inhibitor, designed to selectively target PARP1 (18, 22) and avoid the targeting of PARP2, unlike all the first-generation PARPi. In rat preclinical toxicology models, AZD5305 caused minimal toxicity as monotherapy and improved hematologic tolerability also in combination with carboplatin (CPT; ref. 22). Because of its unique features, AZD5305 opens up multiple options for optimizing the therapeutic window of PARP inhibition in monotherapy and offers new opportunity for combination therapies, particularly with standard-of-care treatments designed to minimize dosage requirements and boost drug efficiency.

Building on the aforementioned rationale, we investigated AZD5305 in a cohort of ovarian cancer patient-derived xenografts (OC-PDXs) representative of *TP53*-mutated high-grade serous (HGS) ovarian cancer (23, 24). We describe the robust *in vivo* anticancer efficacy of AZD5305, a benefit that was particularly evident in *BRCA*-mutated OC-PDXs that were not responsive to the first-generation dual PARP1/2i. In models where PARPi or platinum monotherapies had poor to no effect, AZD5305 in combination with CPT, even at suboptimal doses, stabilized tumor growth, impaired metastatic dissemination and significantly prolonged the lifespan of mice.

## Materials and Methods

### Mice

Six- to 8-week-old Hsd: Athymic Nude-Foxn1nu female mice (Envigo Laboratories) were maintained under specific-pathogen-free conditions, housed in isolated vented cages, and handled using aseptic procedures. Procedures involving animals and their care were conducted in conformity with institutional guidelines that comply with national (Legislative Degree 26/2014) and international (Directive 2010/63/EU) laws and policies, in line with guidelines for the welfare and use of animals in cancer research. Animal studies were approved by the Mario Negri Institute Animal Care and Use Committee and authorized by the Italian Ministry of Health, Directorate-General for Animal Health and Veterinary Medicines (authorization no. 589/2021-PR).

### Drug Preparation and *In Vivo* Dosing

AZD5305 (AstraZeneca) was dissolved in purified water/HCl (pH = 3.5–4) and administered by oral gavage once daily, in cycles of 5 consecutive days followed by 2 days “off drug.” Treatments at 0.1, 1, or 10 mg/kg lasted 8 weeks or were delivered as maintenance (as specified for each experiment).

CPT (Sigma-Aldrich) was dissolved in 0.9% NaCl and injected intravenously once per week for 4 weeks (every 7 days × 4) at doses of 10, 20, 35, or 50 mg/kg (as specified for each experiment).

When given in combination, CPT was administered concurrently with AZD5305 treatments for 4 weeks followed by maintenance treatment with AZD5305 single agent for at least another 4 weeks (as specified for each experiment).

All treatments were well tolerated (Supplementary Fig. S1), including combination therapy with AZD5305 1 mg/kg plus CPT at a dose as high as 50 mg/kg (Supplementary Fig. S1C, right).

### Human Ovarian Adenocarcinoma Xenografts

Five HGS epithelial ovarian cancer patient-derived xenografts (OC-PDXs) were used (Table 1). Each OC-PDX was recovered from cryopreserved stocks and transplanted ectopically or orthotopically.

**TABLE 1:** Characteristics of the OC-PDXs

		Gene alteration^c^		Drug response^d^	
OC-PDX^a^	Engraftment^b^	TP53	BRCA1	Platinum	Olaparib
**HOC22**	Orthotopic	**c.** 993+1G>A	**c.** 1687C>T **p.** Q563*	**S**	**Res**
**HOC84**	Ectopic	**c.** 783–1G>T	wt	**Res**	**Res**
**HOC106**	Ectopic	**c.** 673–1G>C	**c.** 5161delC **p.** Q1721S fs*9	**S**	**S**
**HOC107**	Ectopic	**c.** 517G>A **p.** V173M	**c.** 2727_2730delTCAA **p.** N909K fs*90	**PPS**	**Res**
**HOC520**	Orthotopic	**c.** 920–1G>A	**c.** 4484+5G>A^**&**^ (***r.****4358_4484 del ex13*) **p.** A1453G fs*9	**S**	**Res**

^a^The OC-PDXs were derived from patients diagnosed with stage III/IV HGS ovarian adenocarcinoma relapsed after adjuvant chemotherapy containing platinum, with the exception of HOC520 which was from a treatment naive patient.

^b^Orthotopic: OC-PDX transplanted in the peritoneal cavity (ip). Ectopic**:** OC-PDX growing subcutis (sc).

^c^The genetic alterations were confirmed by Sanger sequencing the OC-PDXs DNA and RNA.

*stop codon. wt: wild type (no mutation detected).

**^&^** The splice donor site variant resulting in the deletion of exon 13 in the RNA (as for RefSeq NM 007294.4).

^d^ **S**: sensitive, **Res**: Resistant, **PPS**: partially platinum-sensitive For the OC-PDXs responsiveness to platinum and olaparib, see tumor growth (subcutaneous) and Kaplan–Meier (intraperitoneal) curves in Supplementary Fig. S2.

### Antitumor Activity

#### Ectopic Setting

Tumor fragments were implanted subcutaneously in the flank of the mice. Tumors were measured with a caliper, and the volume (TV) calculated as [(length × width^2^)/2 = mm^3^]. Mice were randomized (stratified randomization) when tumor volume reached approximately 170–200 mm^3^ (as specified for each experiment) and groups were arbitrarily assigned to the different treatments. Mice were sacrificed when tumor volume reached 1,500 mm^3^ (primary endopoint).

The relative tumor volume was calculated as [TV day *n*/TV at treatment start (day 0)] and the tumor growth curve generated (25). The percentage change in tumor volume was calculated for each individual mouse as [(TV day *n* − TV at day 0)/TV at day 0]*100, and waterfall plots were generated (24). According to RECIST guidelines (26), disease is considered progressive if the tumor volume changes more than + 20%, stable if the change is between + 20% and −30%, regressive if the change is below −30%.

#### Orthotopic Setting

Ascites from donor mice were pooled and injected intraperitoneally as a cell suspension (10 × 10^6^ cells) into the lower right quadrant of the mouse abdomen. OC-PDX–bearing mice were randomized (simple randomization) on the day estimated to correspond to approximately 25%–30% of their life expectancy, based on previous experiments (5 or 10 days posttransplant, respectively for HOC22 and HOC520) and groups were arbitrarily assigned to the different treatments. Mice were monitored daily and were humanely euthanized (marking the limit of survival, primary endpoint) as soon as signs of distress/discomfort, due to disease progression, became apparent (noticeable abdominal effusion, palpable tumor masses). According to the guidelines for animal welfare, disease-related death cannot be an ethical endpoint, so the time to progression (TtP) is recorded as survival time (ST = TtP) and Kaplan–Meier curves are generated (24). For each group, the median ST (MST) was determined and the increment of lifespan (ILS) was calculated as ILS% = 100 × [(treated MST − untreated MST)/untreated MST].

To evaluate the abdominal tumor burden, malignant effusion was harvested and the volume of cancer cells floating in the abdomen recorded. To evaluate metastatic dissemination, the presence of tumor masses in representative organs and anatomic sites such as uterus/ovary, diaphragm, liver, pancreas/omentum, and lymph nodes was rated by two independent scientist using an arbitrary scoring system (0 = no organ infiltration; 1 = small tumor masses; 2 = evident tumor masses; 3 = extensive organ infiltration; 4 = complete organ substitution). The “metastatic score” for each mouse was then calculated as the sum of the rating in all the evaluated organs/anatomic sites (25, 27, 28). Pictures of the peritoneal cavity organs were taken with a macro-digital imaging system (MacroPATH; Milestone S.r.l.).

### Pharmacokinetic and Target Engagement (Pharmacodynamic) Analyses

Plasma and tumor samples were taken 1, 6, and 24 hours after the last of five consecutive doses of AZD5305 (0.1, 1, and 10 mg/kg).

#### Pharmacokinetics

AZD5305 in plasma was analyzed with ultra-performance LC/MS-MS. Each plasma sample was prepared using an appropriate dilution factor and compared against an 11-point standard calibration curve (1–10,000 nmol/L) prepared in DMSO and spiked into blank plasma. To obtain the unbound plasma concentration, the total concentration was multiplied by mouse plasma f_u_ (0.0732) as reported in ref. 18.

### Target Engagement

The pharmacodynamic effect of PARP inhibition by AZD5305 was assessed by Western blotting measuring the reduction of total PARylation in tumor lysates. Tumor samples were lysed in ice-cold buffer containing Tris, NaCl, glycerol, SDS, NP40 (Roche) supplemented with NaF, Na_3_VO_4_, protease complete inhibitor tablet (Roche, 1836145), protease inhibitor cocktail (Sigma, P8340), phosphatase inhibitor cocktails 2 and 3 (Sigma-Aldrich, P0044 and P5726), and benzonase nuclease (Sigma-Aldrich, E1014-5KU). Fastprep tubes and an MP Biomedicals Fast Prep-24 machine were used for homogenization. All samples were sonicated for 30 seconds at high amplitude (Diagenode). Protein concentration was determined using the BCA protein assay kit (Thermo Fisher Scientific). Proteins were separated by SDS-PAGE on NuPAGE 4%–12% Bis-Tris gels (Thermo Fisher Scientific) and transferred onto nitrocellulose membranes using the iBlot dry blotting system. Membranes were blocked with 5% milk in TBS with 0.05% Tween-20 (TBS-T) and incubated overnight at 4°C with primary antibodies (Trevigen, catalog no. 4336-BPC-100, RRID:AB_2721257; Cell Signaling Technology, catalog no. 2118, RRID:AB_561053). Membranes were then washed with TBS-T and incubated with horseradish peroxidase–conjugated anti-rabbit antibody (RRID:AB_2099233) for 1 hour at room temperature. Proteins were detected by incubating membranes with SuperSignal Dura extended-duration substrate (Thermo Fisher Scientific) and visualized using the G:BOX Chemi Genius imaging system (Syngene). Band intensities were quantified using Syngene Genetools software (RRID:SCR_022505).

### Statistical Analysis

Subcutaneous tumor volume was analyzed by ANOVA and Tukey post-test (or *t* test when only two groups were to be compared) every day of measurements. Kaplan–Meier curves were compared by log-rank test. Abdominal tumor burden (malignant ascites and metastatic dissemination) was analyzed by ANOVA and Tukey post-test. Western blot quantification was compared by two-sided Student *t* comparisons on log-transformed data using pooled interindividual variability and adjusted for multiple comparisons by Dunnett test (*, *P* < 0.05; **, *P* ≤ 0.01; ***, *P* ≤ 0.005; ****, *P* < 0.001).

### Data Availability

The data generated in this study are available within the article and its Supplementary Data.

## Results

### AZD5305 has Substantial Antitumor Activity Toward *BRCA-*mutated Subcutaneous Ovarian Adenocarcinoma Xenografts

**FIGURE 1:**
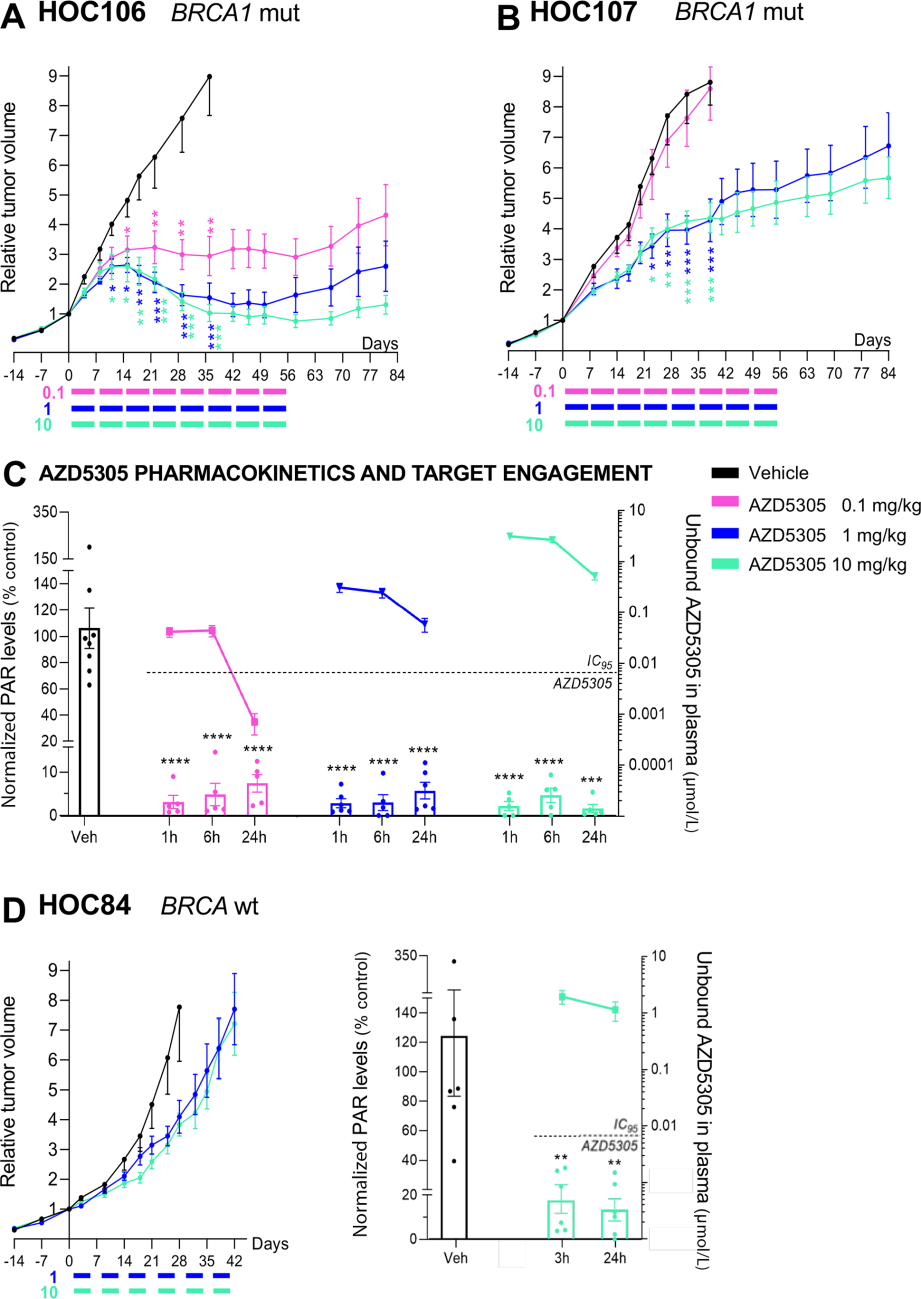
AZD5305 has antitumor activity on subcutaneously growing *BRCA1*m OC-PDXs. **A** and **B,** Relative tumor volume (mean ± SEM) of two *BRCA1*m OC-PDXs: HOC106 (**A**) and HOC107 (**B**). Mice were randomized (stratified randomization) and treated when tumor volume reached 190 mm^3^ (SD 55.9; **A**) or 180 mm^3^ (SD 68.2; **B**). Colored bars indicate the dosing periods: AZD5305 was given orally at 0.1, 1, or 10 mg/kg every day 5 days ON and two OFF. Treatments lasted 8 weeks. Number of mice/group = 6–7. **C,** AZD5305 pharmacokinetics and target engagement at 1, 6, and 24 hours after the last of five consecutive treatments administered to HOC107 tumor-bearing mice. Unbound plasma concentrations of AZD5305 (right axis) and total PARylation levels in the tumor lysates (left axis). **D,***BRCA1*wt HOC84 OC-PDX. Left: AZD5305 efficacy. Relative tumor volume (mean ± SEM). Mice were randomized (stratified randomization) and treated when tumor volume reached 170 mm^3^ (SD 40.8). Colored bars indicate the dosing periods. AZD5305 was given orally at 1 or 10 mg/kg every day 5 days ON and two OFF. Number of mice/group = 7–8. Right: AZD5305 unbound plasma concentrations (right axis) and total PARylation levels in the tumor lysates (left axis) 3 and 24 hours after the last of five consecutive treatments. **A–D,** Statistical significance versus vehicle treated mice was analyzed as specified in Materials and Methods. *, *P* < 0.05; **, *P* ≤ 0.01; ***, *P* ≤ 0.005; ****, *P* < 0.001.

Two subcutaneous *BRCA1*-mutated (*BRCA1*m) OC-PDXs (HOC106 and HOC107) were treated with different doses of AZD5305. In both cases, significant tumor growth inhibition was observed (Fig. 1A and B), regardless of whether they were responsive (HOC106) or not (HOC107) to the first-generation dual PARP1/2 inhibitor olaparib (Supplementary Fig. S2).

AZD5305 affected the growth of HOC106 tumors in a dose-dependent manner (0.1–10 mg/kg) evident after 2 weeks of treatment. AZD5305 0.1 mg/kg led to significant tumor growth delay, while the higher doses (1 and 10 mg/kg), after an initial growth, caused tumor shrinkage. The response was stable for the duration of the treatment (day 56; Fig. 1A). Notably, 4 weeks after therapy stopped (day 84; Fig. 1A) the tumors treated with 10 mg/kg had still not doubled their initial volume.

AZD5305 1 or 10 mg/kg inhibited the growth of HOC107 tumors (Fig. 1B). Compared with the vehicle, a significant reduction in tumor volume was achieved after 3 weeks of treatment and then further improved. Notably, the slower growth rate persisted beyond the treatment period (days 56–84; Fig. 1B). However, 0.1 mg/kg of AZD5305 had no effect.

Interestingly, the antitumor activity of AZD5305 was retained in both HOC106 and HOC107 OC-PDXs pretreated with the dual PARP1/2 inhibitor olaparib (Supplementary Fig. S3).

To test the relationship between AZD5305 efficacy and target engagement, we measured total PARylation in the HOC107 tumors as a pharmacodynamic biomarker. Total PARylation at steady state (after the last of five consecutive daily treatments) was significantly reduced (more than 90% inhibition) by all doses of AZD5305 (≥0.1 mg/kg; Fig. 1C; Supplementary Fig. S4A) indicating complete inhibition of PARP1 for at least 24 hours. Target effective concentrations of AZD5305 were previously defined in relation to duration of exposures above *in vitro* activity against the DLD-1 *BRCA2*^−/−^ cell line (22). Unbound plasma concentrations greater than this target effective concentration of approximately 6 nmol/L were maintained for at least 24 hours by the 1 and 10 mg/kg doses, whereas exposure after 6 hours dropped below this level at the nonefficacious dose of 0.1 mg/kg AZD5305 (Fig. 1C). These data suggest that the *in vivo* preclinical efficacy is associated with sustained and significant reduction (>90%) of PARylation and with maintaining unbound plasma levels of the drug above (>10 folds) the target effective concentration for at least 24 hours.

As a control, to explore the effect of AZD5305 in an HRR-proficient, *BRCA* wild-type (*BRCA*wt) OC-PDX, we examined responses in HOC84, which is not responsive to olaparib nor to platinum treatment (Table 1; Supplementary Fig. S2). As expected, the growth of HOC84 tumors was not affected by 10 mg/kg of AZD5305 (Fig. 1D, left), even though >90% reduction of the tumor's total PARylation was achieved (Fig. 1D, right; Supplementary Fig. S4B) and the unbound plasma levels of AZD5305 remained above 1 μmol/L (∼150 times the IC_95_ in DLD-1 *BRCA2*^−/−^ cells *in vitro* clonogenic assay) for 24 hours. This observation is consistent with PARylation inhibition being necessary but not sufficient for antitumor efficacy, which also requires HRR deficiency.

### AZD5305 Gives a Significant Survival Benefit in Orthotopic Ovarian Adenocarcinoma Xenografts

Next, we studied the effect of AZD5305 in the process of metastatic spread subsequent to debulking surgery. Two OC-PDXs originating from patient's malignant ascites, HOC520 and HOC22, were implanted orthotopically in mice. AZD5305 restrained the malignant progression of these *BRCA1*m OC-PDXs, not responsive to the first-generation dual PARP1/2 inhibitor olaparib (Supplementary Fig. S2).

**FIGURE 2:**
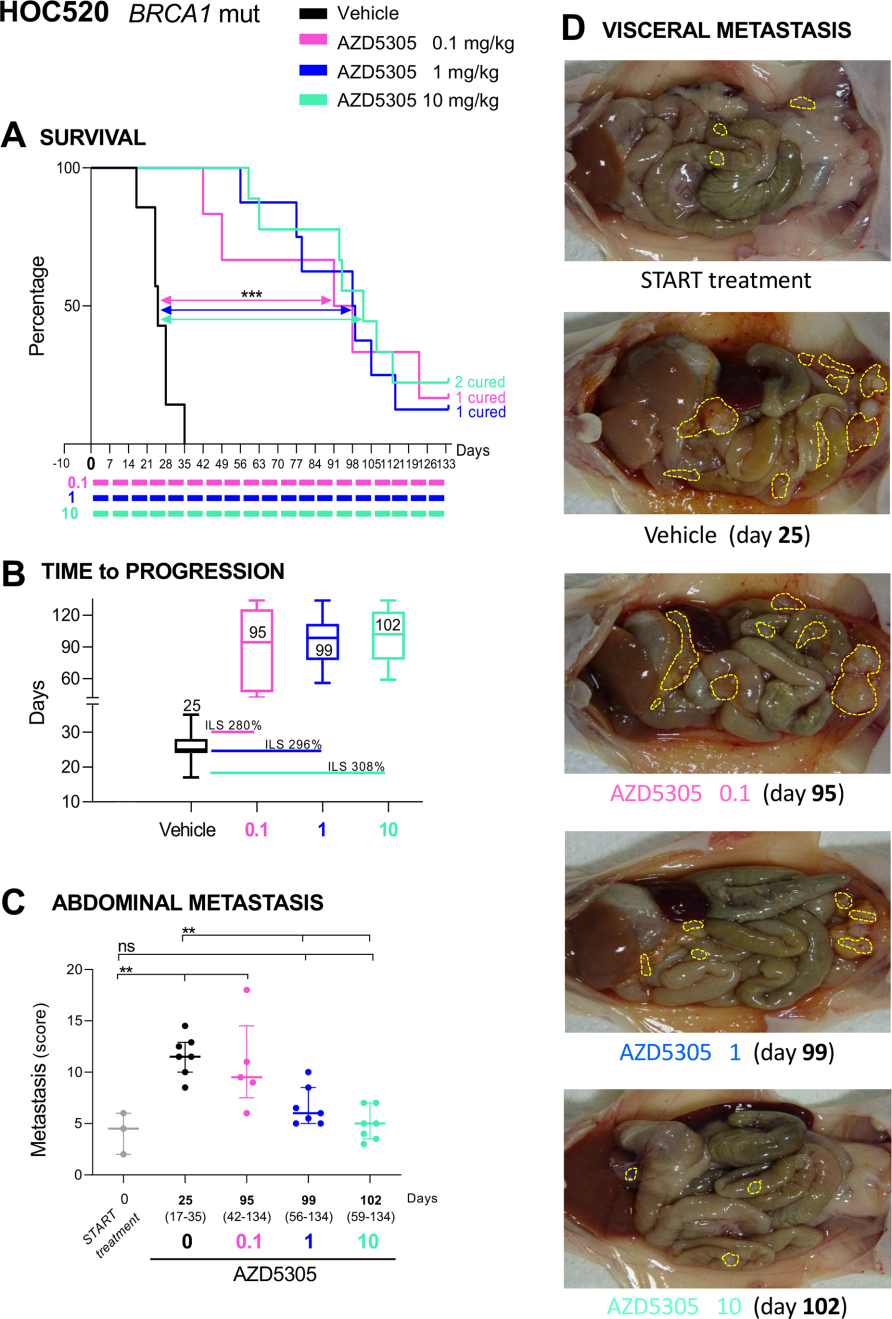
AZD5305 is active on orthotopically growing OC-PDX HOC520, resulting in significant impairment of visceral metastasis and prolonged lifespan of mice. **A,** Kaplan–Meier curves showing the percentage of survival plotted against time elapsed from treatment start. HOC520 OC-PDX–bearing mice were randomized (simple randomization) 10 days after transplantation to receive AZD5305 (0.1, 1, or 10 mg/kg) orally every day 5 days ON and two OFF in maintenance; colored bars indicate the dosing periods. Number of mice/group = 6–9. **B,** Time to progression (TtP). Median with range (days) for each treatment arm; increment of lifespan (ILS%) versus vehicle-treated group is also shown. TtP and ILS% were calculated as described in Materials and Methods. **C,** Abdominal metastasis. Metastatic dissemination (median with interquartile range; scattered points represent the metastasis score of each mouse) at the start of treatment (day 0, *n* = 3) and at disease progression (median with range TtP indicated on the *X* axis). **D,** Representative images of HOC520 visceral dissemination at start of treatments and at time of disease progression (25 days vehicles, 95 days AZD5305 0.1 mg/kg, 99 days AZD5305 1 mg/kg, and 102 days AZD5305 10 mg/kg). **A–C,** Statistical significance versus vehicle treated mice was analyzed as specified in Materials and Methods. ns, not significant; **, *P* ≤ 0.01; ***, *P* ≤ 0.005.

Specifically, the administration of AZD5305 to HOC520 bearing mice (Fig. 2A) resulted in a significant survival benefit at all doses (increment of lifespan % ILS 280, 296, and 308, respectively by 0.1, 1, and 10 mg/kg) compared with the vehicle group (TtP 25 days vs. 95, 99, and 102 days; Fig. 2B). AZD5305 limited the expansion of abdominal metastases in a dose-dependent manner (Fig. 2C and D). The metastatic score in mice treated with 0.1 mg/kg was 9.5 (no different from vehicle-treated mice; score 11.5), whereas in mice treated with 1 or 10 mg/kg it was significantly lower (6 and 5, respectively) and no greater than at the start of treatment (score 4.5), indicating control over tumor growth.

Mice bearing the highly aggressive HOC22 also gained a survival benefit (increment of lifespan, % ILS 114 and 65) upon treatment with 10 and 1 mg/kg of AZD5305, whereas mice given 0.1 mg/kg showed no benefit (Supplementary Fig. S5A and S5B). At the end of study (survival), the abdominal tumor burden was comparable across all the arms (Supplementary Fig. S5C). However, mice treated with 10 mg/kg AZD5305 whose abdominal tumor burden was assessed after 3 weeks of therapy (day 21 interim analysis; Supplementary Fig. S5C, bottom) had significantly less malignant effusion (1.3–1.9 mL) compared with vehicle-treated mice (2.4–4.7 mL) euthanized much earlier (TtP 14 days). This once again indicates that AZD5305 treatment slowed progression of the disease.

On the whole, AZD5305 demonstrated robust antitumor efficacy (reducing tumor growth, containing abdominal dissemination and prolonging survival) in all four *BRCA1*m OC-PDXs tested.

### AZD5305 Sensitizes Ovarian Adenocarcinoma Xenografts to CPT

The benefit of combining AZD5305 and CPT was explored in three *BRCA1*m OC-PDXs, either responsive (HOC106) or not (HOC107 and HOC22) to the dual PARP1/2 inhibitor olaparib and exhibiting different sensitivity to platinum (Table 1; Supplementary Fig. S2).

**FIGURE 3:**
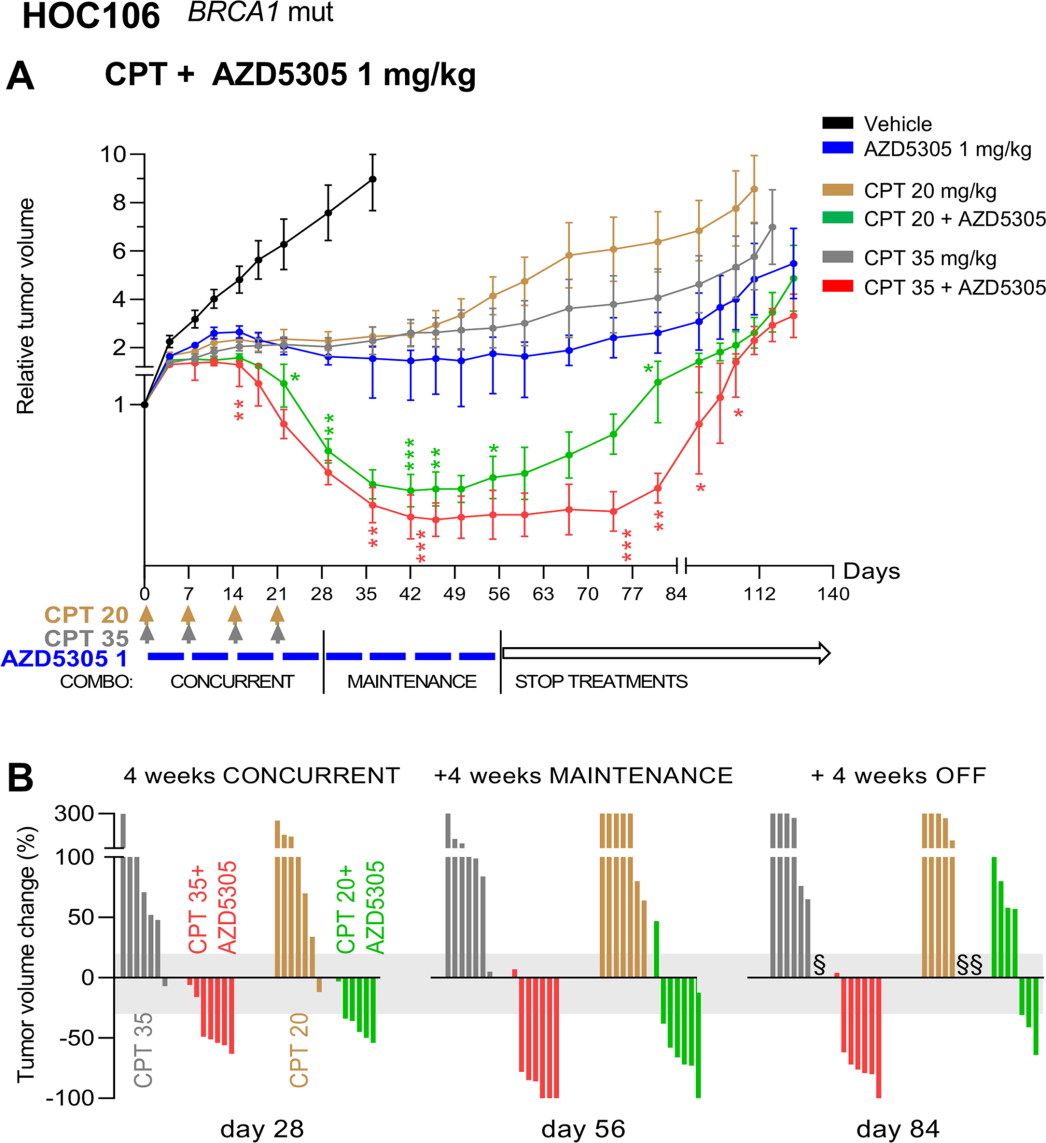
AZD5305 potentiates the effect of CPT, causing significant and sustained regression of HOC106 tumors. **A,** Relative tumor volume (mean ± SEM) of HOC106 tumors growing subcutaneously. Tumor-bearing mice were randomized at a tumor volume of 185 mm^3^ (SD 54.2) to be treated with CPT (20 or 35 mg/kg i.v., once a week for 4 weeks) or AZD5305 (1 mg/kg orally every day, 5 days ON and two OFF for 8 weeks) or combination therapy: 4 weeks of concurrent treatment followed by 4 weeks of AZD5305 single agent (maintenance). Colored bars and arrows indicate the dosing periods. Number of mice/group = 6–7. Statistical significance of the combination versus the matching CPT monotherapy was analyzed as specified in Materials and Methods. *, *P* < 0.05; **, *P* ≤ 0.01; ***, *P* ≤ 0.005. **B,** Treatment efficacy expressed as the percentage of change in tumor volume (compared with the volume at treatment start) after 4 weeks of concurrent treatment (day 28), at the end of the therapy (day 56) and 4 weeks after treatment discontinuation (day 84). Each vertical bar in the waterfall plot represents a single mouse/tumor. Stable disease according to RECIST is highlighted in gray (from +20% to −30%). § Mouse euthanized when the volume considered a predefined endpoint (1,500 mm^3^) was reached.

Single-agent CPT dosed at either 20 or 35 mg/kg and AZD5305 at 1 mg/kg significantly impaired subcutaneous growth of HOC106 tumors, with comparable efficacy across the monotherapy arms (Fig. 3A). The addition of AZD5305 to CPT greatly improved this outcome, with 100% tumors regressing after 4 weeks of therapy in both combination regimens (day 28; Fig. 3B). Tumor regressions were durable (up to day 56) and persisted for at least 4 more weeks after therapy discontinuation in the AZD5305 plus CPT 35 mg/kg treatment arm (day 84; Fig. 3B), while tumors treated with AZD5305 plus CPT 20 mg/kg resumed growth soon after withdrawal of therapy. Nevertheless, the latter combination was still more efficacious than the single-agent CPT at the higher dose of 35 mg/kg (Fig. 3A) and comparable with CPT 35 mg/kg combined with a lower dose of AZD5305 (0.1 mg/kg; Supplementary Fig. S6), where combination was significantly superior to either monotherapy.

**FIGURE 4:**
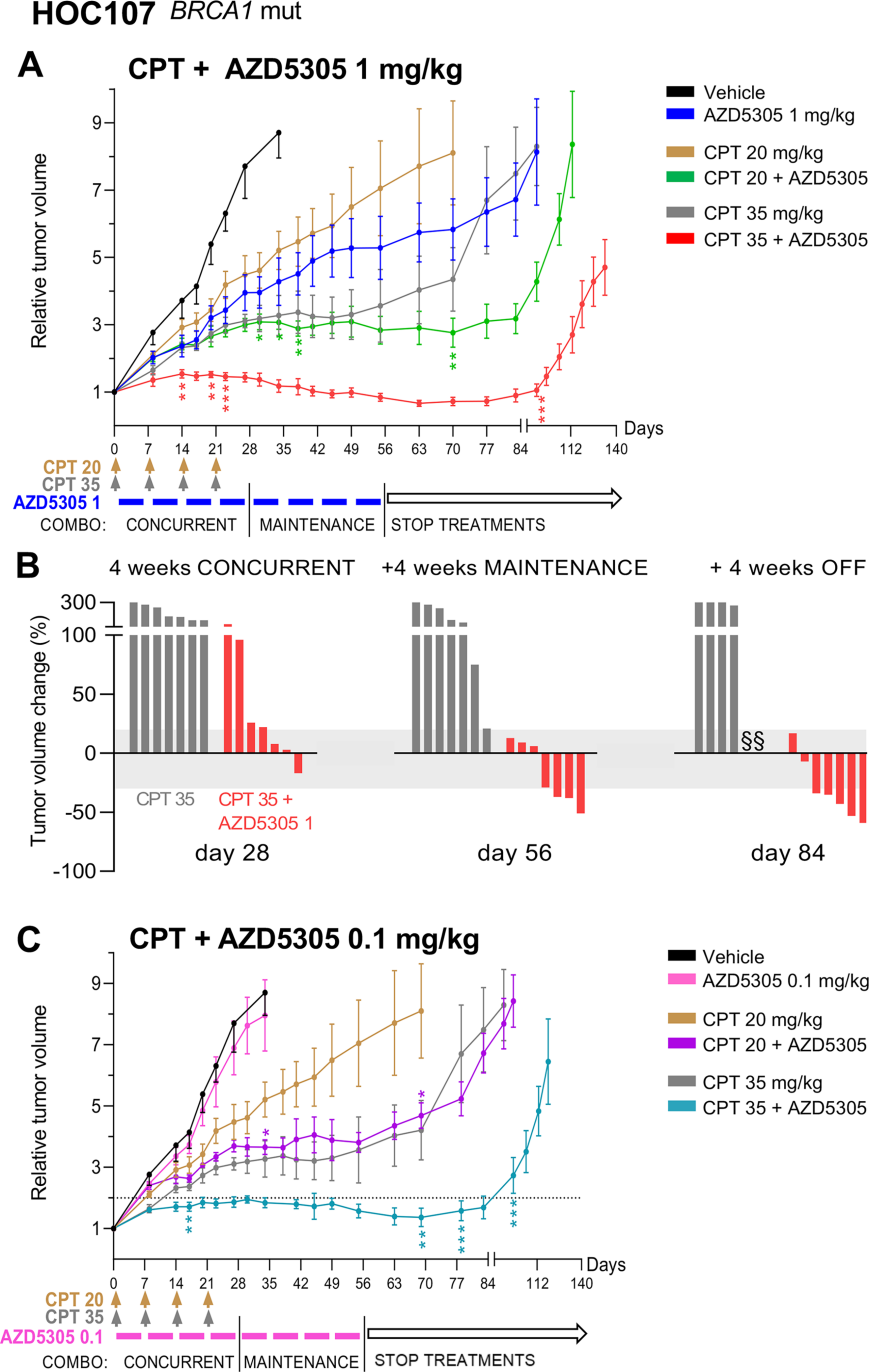
AZD5305 potentiates the effect of CPT, significantly impairing the growth of HOC107 tumors. **A,** Relative tumor volume (mean ± SEM) of HOC107 tumors growing subcutaneously. Tumor-bearing mice were randomized at a tumor volume of 200 mm^3^ (SD 67.3) and were treated with CPT (20 or 35 mg/kg i.v., once a week for 4 weeks) and/or AZD5305 1 mg/kg orally every day, 5 days ON and two OFF for 8 weeks, or combination therapy: 4 weeks of concurrent treatment followed by 4 weeks of AZD5305 single agent (maintenance). Colored bars and arrows indicate the dosing periods. Number of mice/group = 6–7. **B,** Efficacy of the combination CPT plus AZD5305 1 mg/kg expressed as the percentage of change in tumor volume (compared with the volume at treatment start) after 4 weeks of concurrent treatment (day 28), at the end of the therapy (day 56) and 4 weeks after treatment discontinuation (day 84). Each vertical bar in the waterfall plot represents a single mouse/tumor. Stable disease according to RECIST is highlighted in gray (change from +20% to −30%). § Mouse euthanized when the volume considered a predefined endpoint (1,500 mm^3^) was reached. **C,** Relative tumor volume (mean ± SEM) of HOC107 tumors growing subcutaneously (vehicle and CPT single agent are the same arms as in **A**). Tumor-bearing mice were randomized and were treated with CPT (20 or 35 mg/kg) and/or AZD5305 0.1 mg/kg or combination therapy (as described in **A**). Colored bars and arrows indicate the dosing periods. Number of mice/group = 6–7. Statistical significance of the combination versus matching CPT monotherapy was analyzed as specified in Materials and Methods. *, *P* < 0.05; **, *P* ≤ 0.01; ***, *P* ≤ 0.005.

Single-agent CPT inhibited HOC107 tumor growth in a dose-dependent manner. The CPT plus AZD5305 combination therapies outperformed the single-agent treatments and the benefits varied according to the dosing. Specifically, AZD5305 1 mg/kg (Fig. 4A) added to 20 mg/kg CPT significantly potentiated the antitumor activity: the impairment of tumor growth was comparable with that of CPT monotherapy dosed higher at 35 mg/kg but lasted significantly longer. When AZD5305 was added to 35 mg/kg CPT, the combination was significantly more effective: at the end of treatment (day 56; Fig. 4B) all of the tumors (7/7) had an objective response (either stable disease or regression). The effect was durable and lasted for additional 4 weeks after therapy discontinuation (day 84; Fig. 4B), and none of the tumors had increased their size >20%.

CPT 35 mg/kg combined with a lower dose of AZD5305 (0.1 mg/kg which was not efficacious as monotherapy) resulted in durable tumor growth inhibition (Fig. 4C): 4 weeks after therapy discontinuation (day 84), HOC107 tumors had still not doubled their initial volume. AZD5305 0.1 mg/kg also significantly potentiated the antitumor activity of CPT 20 mg/kg, with efficacy equal to that of the single-agent CPT at the higher dose of 35 mg/kg (Fig. 4C).

**FIGURE 5:**
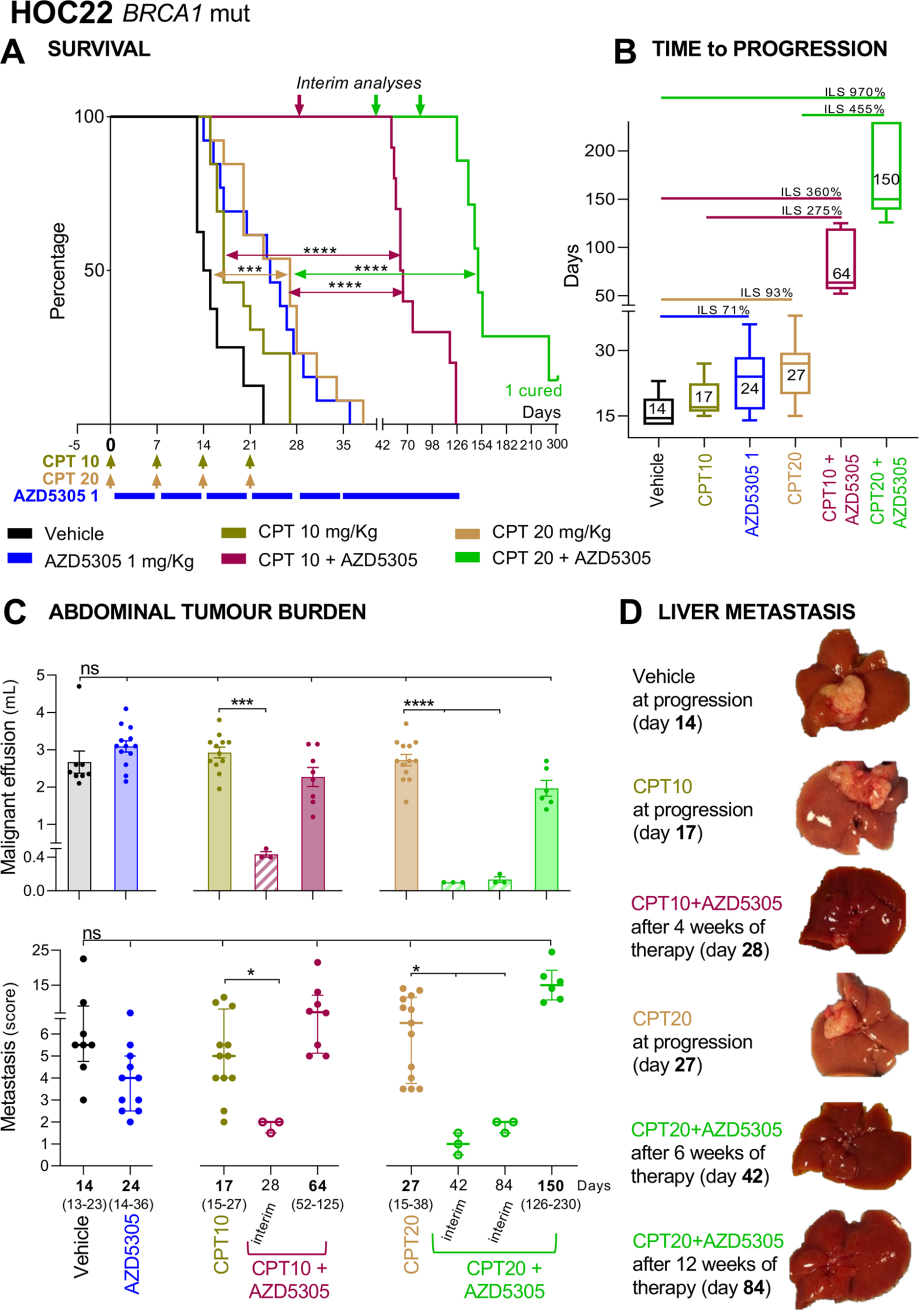
AZD5305 plus CPT significantly impairs the progression of the abdominal disease limiting metastasis and prolonging the lifespan of HOC22 bearing mice. **A,** Kaplan–Meier curves showing the percentage of survival plotted against time elapsed from treatment start. HOC22 bearing mice were randomized (simple randomization) 5 days after transplantation to receive CPT at 10 or 20 mg/kg (i.v. once a week for 4 weeks) and AZD5305 dosed at 1 mg/kg (orally every day 5 days ON and two OFF, as maintenance regimen) or combination therapy: 4 weeks of concurrent treatment followed by AZD5305 single agent. Colored bars and arrows indicate the dosing. Number of mice/group = 13 (vehicle *n* = 8). **B,** Time to progression (TtP). Median with range (days) for each treatment arm; increment of lifespan (ILS%) is also shown. TtP and ILS% were calculated as described in Materials and Methods. **C,** Abdominal tumor burden. Malignant effusion (top: volume: mean ± SEM) and metastatic dissemination to the peritoneal organs (bottom: median with interquartile range; scattered points are the metastasis scores for each mouse) at time of disease progression (median with range TtP indicated on the *X* axis) for each treatment arm and at predefined interim times during combination therapy (at days 28, 42, and 84, *n* = 3). **D,** Representative images of disseminated livers at time of disease progression (14 days vehicles, 17 days single-agent CPT at 10 mg/kg, and 27 days single-agent CPT at 20 mg/kg) and at predefined interim times during combination therapy, specifically after 4 weeks (day 28) for AZD5305 plus CPT 10 mg/kg, and after 6 and 12 weeks (days 42 and 84) for AZD5305 plus CPT 20 mg/kg. **A** and **C,** Statistical significance as specified in Materials and Methods. ns, not significant; *, *P* < 0.05; ***, *P* ≤ 0.005; ****, *P* < 0.001.

To reinforce the translational value of the findings, mice were orthotopically implanted with HOC22 (Fig. 5), an OC-PDX that mimics human disease with dissemination in the mouse peritoneal cavity. This OC-PDX is very responsive to platinum (Supplementary Fig. S2), so for this study, suboptimal dosing of CPT was used. Disease-bearing mice treated with single-agent CPT 20 mg/kg gained significantly prolonged survival (% ILS 93) compared with the vehicle-treated mice, whereas CPT 10 mg/kg gave no benefit (% ILS 20; Fig. 5A and B).

The addition of 1 mg/kg AZD5305 (% ILS 71 as single agent) to 10 mg/kg CPT significantly prolonged survival (% ILS 360; Fig. 5A) and also performed significantly better than single-agent CPT 20 mg/kg (Fig. 5A and B). Most importantly, the combination outperformed the gain in survival achieved by the full-dose platinum treatment: CPT 35 mg/kg and cisplatin 4 mg/kg (% ILS 200 and 250, respectively; Supplementary Fig. S2).

The benefit of adding AZD5305 to CPT 10 mg/kg was already evident after 4 weeks of treatments (day 28 interim analysis; Fig. 5C and D), at which time both abdominal metastases and malignant effusion were significantly less (metastasis score 2 and malignant ascites 0.4 mL) than in either single agent–treated mice (metastasis score ∼5 and malignant ascites 3 mL) which had to be euthanized much earlier (TtP 17 or 24 days, respectively for CPT or AZD5305). These are strong indications that the combination delayed the spread of the disease throughout the organs of the peritoneal cavity, and in fact the time for the disease to progress to a comparable abdominal burden was significantly longer (TtP 64; Fig. 5B).

Combination of 20 mg/kg of CPT plus 1 mg/kg AZD5305 significantly prolonged survival (% ILS 970; Fig. 5A). After 6 and 12 weeks on therapy (days 42 and 84 interim analysis; Fig. 5C and D), mice did not present any signs of distress yet, and had a reduced amount of abdominal metastasis (metastasis score 1–2) and malignant effusion (0.2 mL). As a consequence, combination-treated mice lived significantly longer (TtP 150 days; Fig. 5B) than CPT 20 mg/kg single agent–treated mice (TtP 27 days) taken off study because of the severe abdominal tumor burden (metastasis score 6.5 and malignant ascites 3 mL).

Collectively, the results indicate that the addition of AZD5305 to CPT drove superior efficacy in three *BRCA1*m OC-PDXs regardless of their innate responsiveness to platinum and/or to olaparib monotherapies. Conversely, in the case of HOC84, the *BRCA*wt OC-PDX resistant to AZD5305 (Fig. 1D), CPT and cisplatin (Supplementary Fig. S2), limited benefit was attained by the combination therapy (CPT 50 mg/kg plus AZD5305 1 mg/kg; Supplementary Fig. S7) suggesting that tumors may need to have some innate sensitivity to either monotherapy to benefit from the combination.

## Discussion

We report that AZD5305 has strong antitumor activity as a single agent in preclinical models of ovarian cancer and is an efficacious partner with CPT. The effects were demonstrated across a cohort of *TP53*-mutated OC-PDXs derived from HGS ovarian adenocarcinoma [HGSOC (23), the subtype with the highest death rate (29, 30)], differing in their innate responsiveness to platinum.

AZD5305 was most effective (tumor growth reduction, abdominal dissemination containment and survival gain) in OC-PDXs with underlying DNA repair deficits, such as deleterious mutations in *BRCA1*. This occurred even though three (out of four) of these PARPi-naïve OC-PDXs (HOC22, HOC107, and HOC520) were resistant to first-generation dual PARP1/2 inhibitors such as olaparib, and also in OC-PDXs pretreated with olaparib (HOC106 and HOC107).

In addition to affecting tumor growth, AZD5305 as single-agent significantly impaired tumor dissemination (visceral metastasis) and prolonged the lifespan of mice bearing OC-PDXs implanted orthotopically to mimic the progressive disease in patients after debulking surgery (25). This effect on the metastatic setting, which is responsible for the greatest morbidity and mortality for patients with ovarian cancer, is particularly noteworthy in relation to the potential clinical utility of AZD5305 for this tumor.

The first generation of PARPi, such as olaparib, niraparib, and rucaparib, have become the therapeutic choices for HGSOC (4). The currently approved PARPi are essentially equipotent against PARP1 and PARP2 enzymes (19). It has been suggested that the PARP2 function is not essential for antitumor activity in HRR-deficient cancer models, and that only PARP1 inhibition is required (20). Fittingly, our results not only demonstrate that AZD5305 [a selective PARP1 inhibitor and trapper (18)] is very effective, but also provide a strong preclinical rationale for its use in alternative to therapy with the first-generation dual PARP1/2 inhibitors. This efficacy is most notable at doses of 1 or 10 mg/kg AZD5305, where sustained target inhibition is maintained above target-effective concentrations for >24 hours. In contrast, olaparib treatment at 100 mg/kg did not maintain target inhibition and exposures above target effective concentration for 24 hours (Supplementary Fig. S8), which may contribute to its reduced efficacy in less PARPi-sensitive models.

Combination strategy is an area of great interest, aiming to enhance the anticancer effects of PARP inhibition and the companion agents, thus expanding the patient population eligible for therapy. The combination of PARPi with antiangiogenic drugs is well tolerated and clinically effective (7, 31, 32) and the use of olaparib plus bevacizumab has recently obtained regulatory approval (9, 10). Combinations with chemotherapy are under investigation too (11–14), but their use is not currently approved (33). Hematologic toxicities limit the clinical applicability of PARPi combined with cytotoxic chemotherapy because of dose-limiting cytopenias (34, 35). Thus the combination of PARPi with chemotherapy remains therapeutically promising but challenging and PARPi selective for PARP1 could expand the potential therapeutic strategies. PARP2 may be in fact associated with hematologic toxicity (21) while efficacy may be solely driven by inhibition and trapping of PARP1 (19, 20). Accordingly, in rat preclinical toxicology models, AZD5305 caused minimal hematologic toxicity as monotherapy and better hematologic tolerability than first-generation dual PARP1/2 inhibitors also in combination with CPT (22).

In the current study, the addition of AZD5305 to CPT impaired tumor growth, controlled disease progression, and prolonged the lifespan of mice better than either drug as single agent, highlighting the potential for its use in combination. A few preclinical scenarios are worth commenting upon.

First, the combination was more effective in *BRCA*1m OC-PDXs not responsive to the first-generation PARPi, and the greater efficacy was evident even at doses of AZD5305 that had little (HOC22) or no effect (HOC107) singly.

Second, in respect to the highly aggressive platinum-sensitive HOC22, the greater efficacy (impaired metastatic dissemination, prolonged lifespan of diseased mice) was particularly evident when CPT was used at suboptimal doses, which in themselves were poorly or not effective, and the combinations were superior even to full-dose platinum. These findings suggest that lowering the dose of platinum may still provide a therapeutic benefit in combination, while potentially reducing systemic toxicity.

Third, one of the issues in the clinical setting is the limited treatment options for partially platinum-sensitive HGSOC (i.e., patient initially platinum sensitive, but relapsing between 6 and 12 months). The combination of CPT with AZD5305 gave a good antitumor response even against the poorly platinum-responsive HOC107 tumors, an OC-PDX from a patient who relapsed sooner than 12 months after adjuvant chemotherapy containing platinum (23).

These results support the use of AZD5305 combined with CPT, with close attention to dose and schedule. Clinical trials in humans will answer whether this regimen is tolerated and effective.

While AZD5305 markedly improved outcomes in all the *BRCA1m* OC-PDXs tested, whether sensitive or not to olaparib, it had no activity in the *BRCAwt* tumor tested. Despite good plasma exposure and on-target molecular effect (target engagement as a result of treatment) *BRCA*wt HOC84 did not benefit from therapy, indicating that the genetic context must be considered, as with the first-generation PARPi. Moreover, the PARP inhibition by AZD5305 was not enough to overcome the intrinsic platinum resistance of HOC84. The rationale for adding AZD5305 to CPT is to boost the anticancer potential by reducing the cancer cells’ ability to repair the DNA damage. We speculate that CPT monotherapy in HOC84 may have been insufficient to cause DNA damage, and hence there was no enhancement or efficacy by PARP inhibition. Our results are in line with the general view that tumors need to have some response to CPT as single agent, to be most responsive to the combination with AZD5305.

In conclusion, AZD5305, a novel selective PARP1 inhibitor and trapper, shows significant antitumor activity toward *BRCA1*m OC-PDXs resistant to olaparib, and potentiated the therapeutic efficacy of CPT also toward partially platinum-sensitive OC-PDXs. The use of this drug alone or in combination is reinforced by the benefit in controlling disease progression in OC-PDXs that mimic human disease (reduction of visceral dissemination and malignant ascites), resulting in significant prolongation of the lifespan of mice.

AZD5305 is currently in phase I clinical trials. Our preclinical results provide a strong rationale for this clinical investigation that foresee combination with CPT as a potential means of maximizing clinical benefit and minimizing dosage requirements while increasing efficiency.

## Supplementary Material

Supplementary Fig. S1 Figure S1 shows absence of drug-related toxicity

Supplementary Fig. S2 Figure S2 shows the response of the OC-PDXs to platinum and olaparib

Supplementary Fig. S3 Fig. S3 shows results of the "switching" therapy

Supplementary Fig. S4 Figure S4 shows AZD5305 target engagement

Supplementary Fig. S5 Fig. S5 shows the effect of AZD5305 on the OC-PDX HOC22

Supplementary Fig. S6 Fig. S6 shows the effect of the combination with suboptimal doses of CPT or AZD5305

Supplementary Fig. S7 Fig. S7 shows the effect of the combination on OC-PDX HOC84

Supplementary Fig. S8 Fig. S8 shows olaparib pharmacokinetics and target engagement
