# Supplementary material for: The PARP1 Inhibitor AZD5305 Impairs Ovarian Adenocarcinoma Progression and Visceral Metastases in Patient-derived Xenografts Alone and in Combination with Carboplatin
Source: Cancer Res Commun. 2023 Mar 27;3(3):489–500. doi: 10.1158/2767-9764.CRC-22-0423 (PMC10042207; doi:10.1158/2767-9764.CRC-22-0423)
Supplement: Supplementary Fig. S1 — Figure S1 shows absence of drug-related toxicity [file crc-22-0423-s01.pdf]

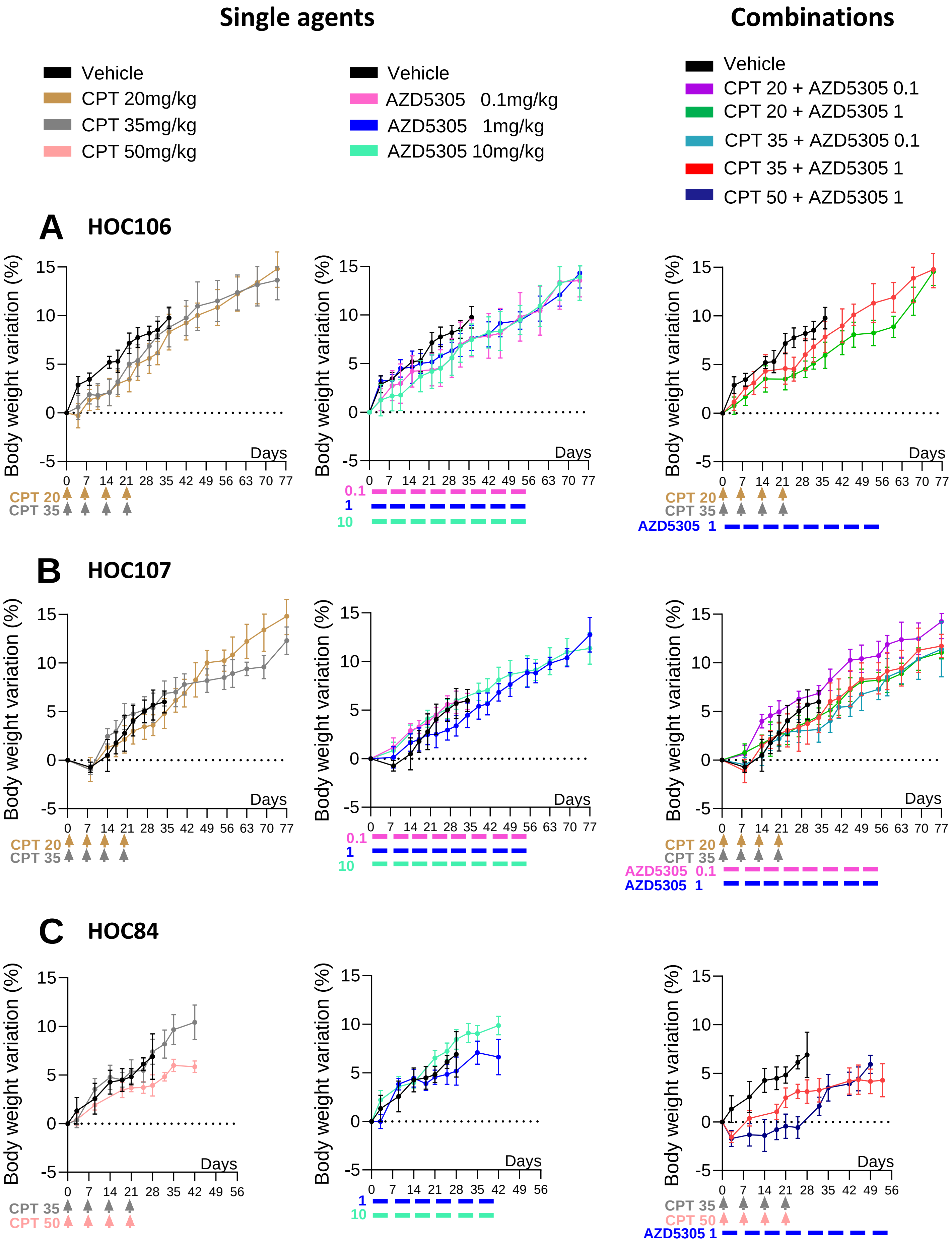

Supplementary Figure S1

(see legend on next page)

Supplementary Figure S1

Changes in mice body weight as a rough indication of drug-related toxicity.

A-C. Body weight was monitored over time and changes from the beginning of treatment calculated as 100*[(weight day n - weight day 0)/ weight day 0] and plotted (mean ± SEM).

A HOC106, **B** HOC107 and **C** HOC84 receiving carboplatin (CPT) intravenously once a week for 4 weeks (**left**), AZD5305 orally once a day (5 days ON/2 OFF) for 8 weeks (**middle**), or the two drugs (4 weeks of concurrent treatment followed by 4 weeks of AZD5305 single-agent, **right**)**.** Number of mice/group=6-8.

Therapies were well tolerated and body weight loss never exceeded 5%, even when AZD5305 1 mg/kg was combined with CPT at doses as high as 50 mg/kg.
