# Supplementary material for: The PARP1 Inhibitor AZD5305 Impairs Ovarian Adenocarcinoma Progression and Visceral Metastases in Patient-derived Xenografts Alone and in Combination with Carboplatin
Source: Cancer Res Commun. 2023 Mar 27;3(3):489–500. doi: 10.1158/2767-9764.CRC-22-0423 (PMC10042207; doi:10.1158/2767-9764.CRC-22-0423)
Supplement: Supplementary Fig. S2 — Figure S2 shows the response of the OC-PDXs to platinum and olaparib [file crc-22-0423-s02.pdf]

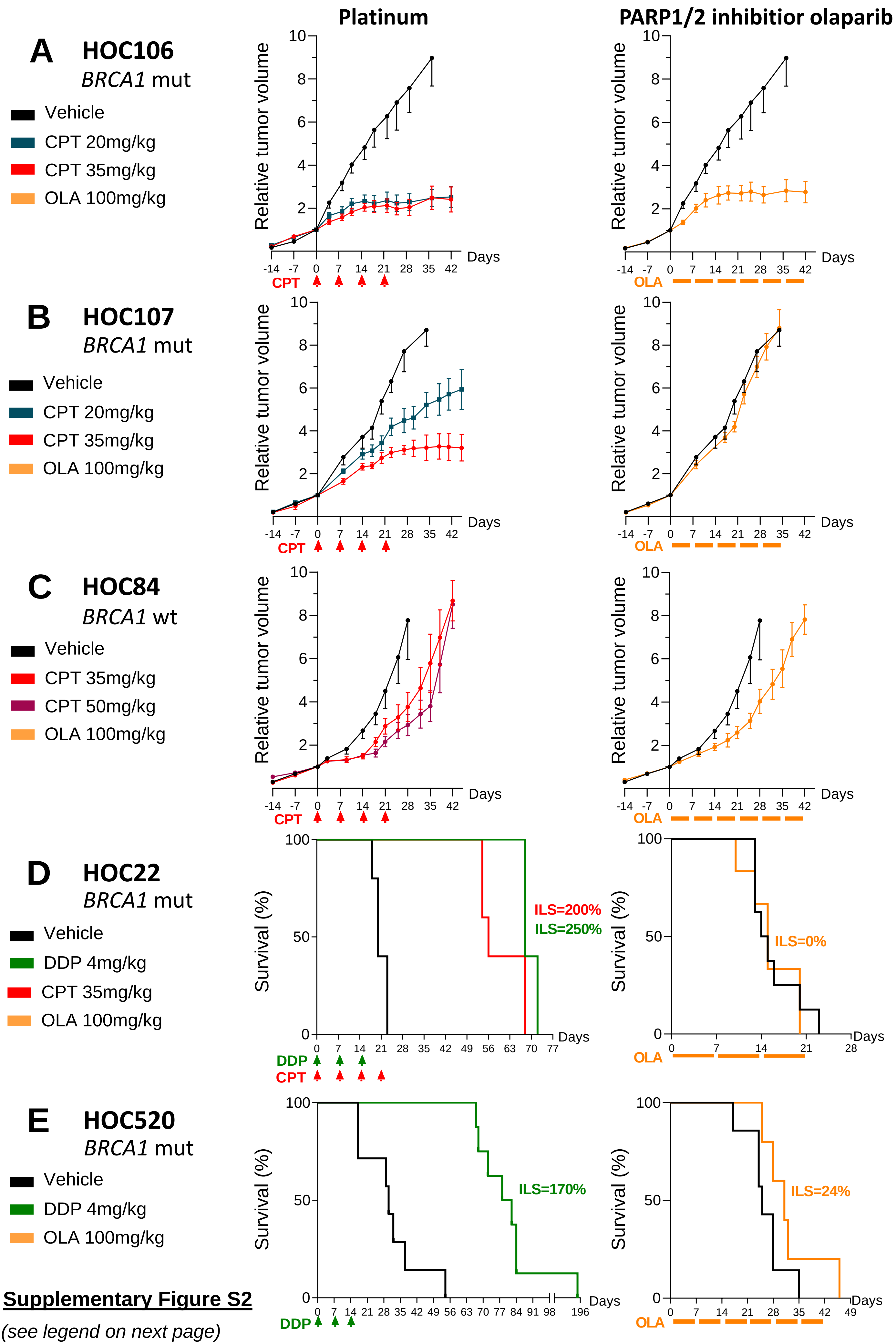

Supplementary Figure S2

(see legend on next page)

Supplementary Figure S2

Responsiveness of the OC-PDXs to platinum (left) and first-generation dual PARP1/2 inhibitor olaparib (right).

A-E. Colored bars and arrows indicate the dosing periods.

Carboplatin (CPT, Sigma-Aldrich) was dissolved in 0.9% NaCl and injected intravenously (iv) once a week for 4 weeks (Q7x4) at the doses of 20, 35 or 50 mg/kg (as indicated).

Cisplatin (DDP, cis-diaminedichloroplatinum, Sigma Aldrich) was dissolved in 0.9% NaCl and injected intravenously (iv) at 4 mg/kg once week for 3 weeks (Q7x3).

Olaparib (OLA, AZD2281, AstraZeneca) was formulated in 10% v/v DMSO in 30% w/v Kleptose (HP-β-CD) in sterile MilliQ water and given orally at 100 mg/kg once daily (QD) for 5 days ON and 2 OFF.

A-C. Anti-tumor activity towards OC-PDXs growing subcutaneously, presented as relative tumor volume (mean ± SEM). Number of mice/group=6-8. HOC106 (*BRCA1*m) is sensitive to both CPT and OLA, and both drugs stalled tumor growth (**A**). HOC107 (*BRCA1*m) is partially sensitive to CPT (with different degrees of response depending on the dose) and resistant to OLA (**B**). HOC84 (*BRCA*wt) is resistant to both CPT and OLA (**C**).

D-E. Activity towards OC-PDXs growing orthotopically, presented as Kaplan Meier curves (number of mice/group=6-8). The lifespan increment (ILS% calculated as described in Materials and Methods) of disease-bearing mice is shown. Despite being *BRCA1*m, both HOC22 (**D**) and HOC520 (**E**) are resistant to OLA but sensitive to platinum (DDP or CPT) which significantly prolonged the lifespan of mice.
