# Supplementary material for: The PARP1 Inhibitor AZD5305 Impairs Ovarian Adenocarcinoma Progression and Visceral Metastases in Patient-derived Xenografts Alone and in Combination with Carboplatin
Source: Cancer Res Commun. 2023 Mar 27;3(3):489–500. doi: 10.1158/2767-9764.CRC-22-0423 (PMC10042207; doi:10.1158/2767-9764.CRC-22-0423)
Supplement: Supplementary Fig. S3 — Fig. S3 shows results of the "switching" therapy [file crc-22-0423-s03.pdf]

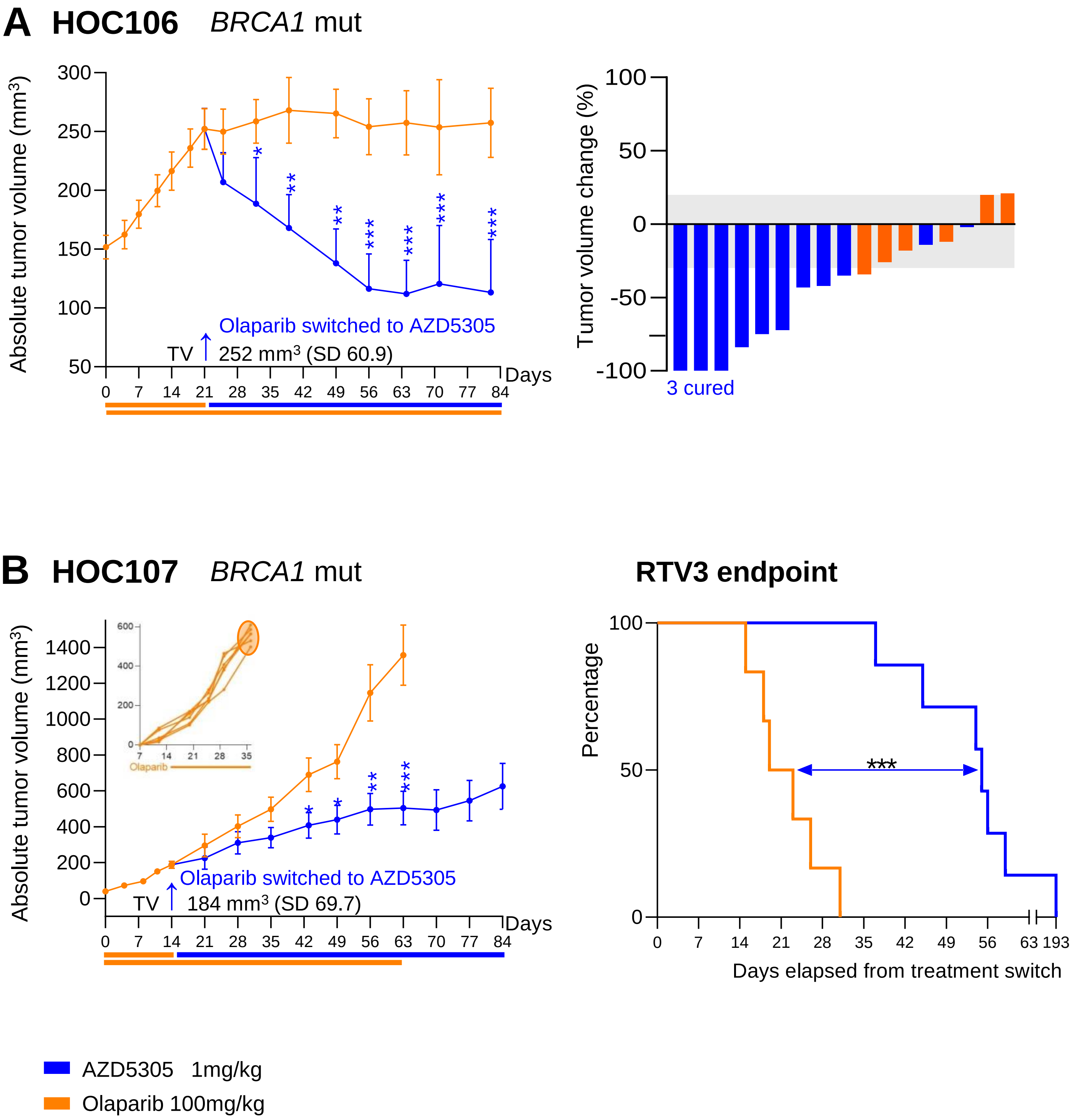

Supplementary Figure S3

(see legend on next page)

Supplementary Figure S3

AZD5305 preserves greater efficacy against tumors previously treated with first-generation dual PARP1/2 inhibitor olaparib.

A and **B (left). Absolute tumor volume** (mean ± SEM) of the *BRCA1*m OC-PDXs HOC106 (**A**) and HOC107 (**B**). Colored bars indicate the dosing periods. Olaparib 100 mg/kg or AZD5305 1 mg/kg were given orally five days ON and two OFF.

A. **HOC106** tumor bearing mice were randomized at a tumor volume of 150 mm^3 (SD 35.0), treated for 3 weeks with olaparib and then randomized again at a tumor volume of 252 mm^3 (SD 60.9) to be treated with AZD5305 (switching therapy, n=11) or continue with olaparib (n=6). Shown on the right is the treatment efficacy expressed as the percentage volume change after 8 weeks of treatment (day 84) compared with the volume when therapy switched (day 21). Each vertical bar in the waterfall plot represents a single mouse/tumor.

Nine out of 11 (82%) HOC106 tumors challenged by switching to AZD5305 regressed (according to RECIST criteria) whereas olaparib-treated tumors remained stable (+20 to -30 %).

B. **HOC107** tumors underwent olaparib therapy for five weeks (donor mice; reported in the insert at the side), prior being transplanted in investigational mice and treated with olaparib for further 2 weeks and then randomized at a tumor volume of 184 mm^3 (SD 69.7) to be treated with AZD5305 (switching therapy, n=7) or continue with olaparib (n=7). The time taken to reach 3 times the tumor volume (RTV3) was calculated and is shown on the right.

HOC107 tumors challenged by switching to AZD5305 grew significantly more slowly than tumors continuing with olaparib, with a significant difference in the time taken to achieve a 3-fold increase in volume.

A-D Statistical significance assessed as specified in Materials and Methods.

* $p<0.05$; ** $p\leq0.01$; *** $p\leq0.005$
