# Supplementary material for: The PARP1 Inhibitor AZD5305 Impairs Ovarian Adenocarcinoma Progression and Visceral Metastases in Patient-derived Xenografts Alone and in Combination with Carboplatin
Source: Cancer Res Commun. 2023 Mar 27;3(3):489–500. doi: 10.1158/2767-9764.CRC-22-0423 (PMC10042207; doi:10.1158/2767-9764.CRC-22-0423)
Supplement: Supplementary Fig. S4 — Figure S4 shows AZD5305 target engagement [file crc-22-0423-s04.pdf]

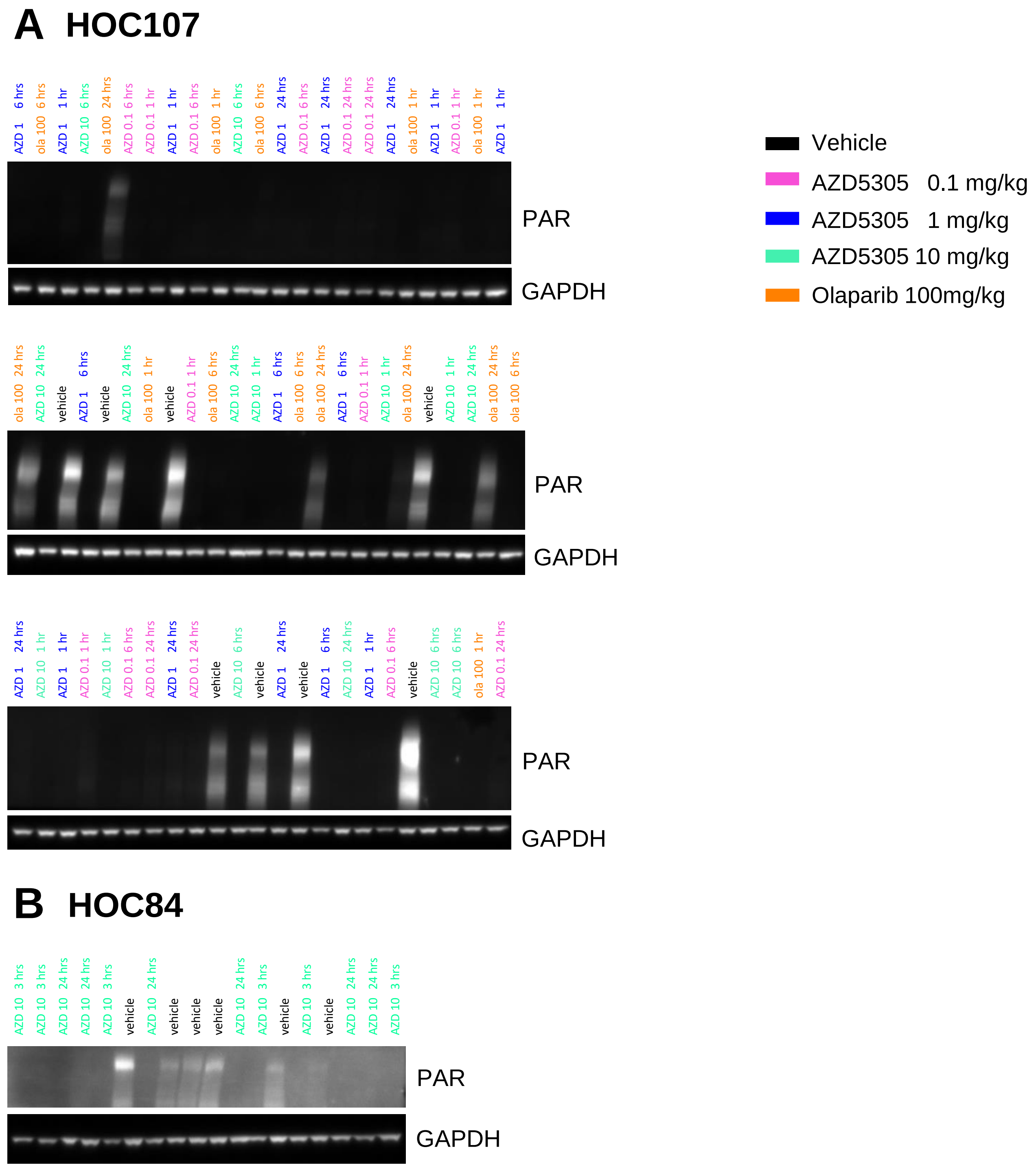

Supplementary Figure S4: AZD5305 target engagement

Western Blot of PAR protein in HOC107 and HOC84 tumor samples. GAPDH was used as internal control. Samples are loaded in randomized order in order to remove bias or gel loading artifacts to aid in the accuracy of the quantitation process.

A. HOC107 tumor samples, collected 1, 6 and 24 hrs after five consecutive AZD5305 (0.1, 1, 10 mg/kg) or Olaparib (100mg/kg) treatments. Total PARylation was significantly reduced by all doses of AZD5305 indicating complete inhibition of PARP1 for at least 24 h (see quantification Fig.1C). On the contrary, PAR levels recovered after 6 h following olaparib treatment (see quantification Supplementary Fig.S8).

B. HOC84 tumor samples, collected 3 and 24 hrs after five consecutive AZD5305 (10 mg/kg) treatments. A significant reduction of PARylation was observed for at least 24h (see quantification Figure 1D), indicating that AZD5305 lack of anti-tumor activity is not linked to ineffective target engagement.
