# Supplementary material for: The PARP1 Inhibitor AZD5305 Impairs Ovarian Adenocarcinoma Progression and Visceral Metastases in Patient-derived Xenografts Alone and in Combination with Carboplatin
Source: Cancer Res Commun. 2023 Mar 27;3(3):489–500. doi: 10.1158/2767-9764.CRC-22-0423 (PMC10042207; doi:10.1158/2767-9764.CRC-22-0423)
Supplement: Supplementary Fig. S5 — Fig. S5 shows the effect of AZD5305 on the OC-PDX HOC22 [file crc-22-0423-s05.pdf]

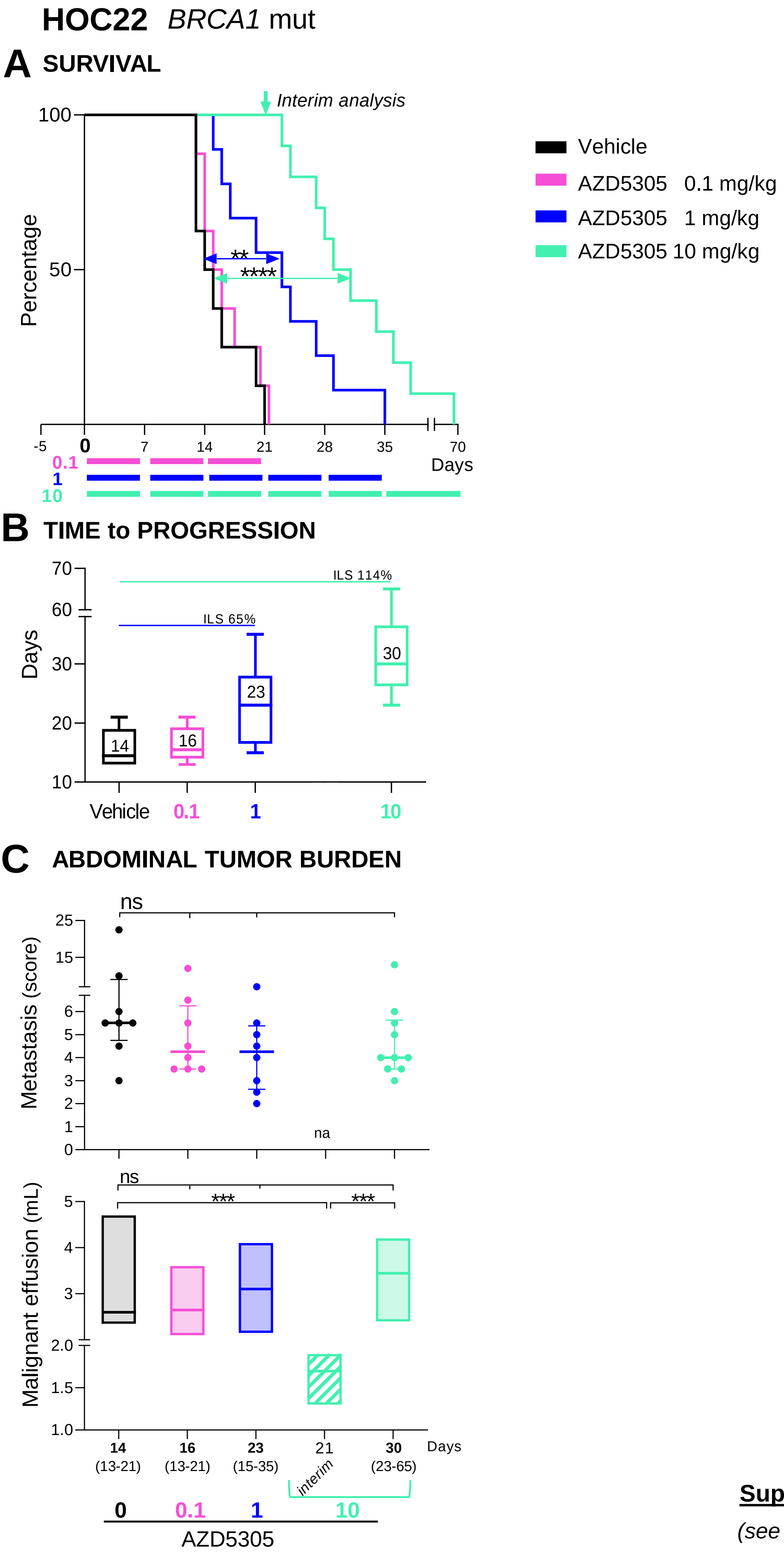


**Supplementary Figure S5**

*(see legend on next page)*

**Supplementary Figure S5**

**AZD5305 is efficacious in orthotopic OC-PDX HOC22 significantly prolonging the lifespan of mice.**

**Kaplan Meier curves** showing the percentage of survival over time elapsed from treatment start. HOC22 bearing mice were randomized (simple randomization) 5 days after transplantation to receive AZD5305 (0.1, 1 or 10 mg/kg) given orally QD, five days ON and two OFF in maintenance; colored bars indicate the dosing periods. Number of mice/group=8-10

**B. Time to disease progression** (TtP). Median with range (days) is shown for each treatment arm; increment of life span (ILS%) *vs* vehicle-treated group is also shown. TtP and ILS were calculated as described in Materials and Methods.

**C. Abdominal tumor burden** at disease progression, TtP (median with range) for each group indicated on the X axis. **Upper panel**: metastatic dissemination (median with interquartile range; scattered points representing the metastasis score for each mouse; na=not available). **Lower panel**: malignant effusion (amount of cancer cells in the abdomen: volume min to max with line at median).

Shown is also the malignant effusion of mice treated with 10 mg/kg AZD5305 whose abdominal tumor burden was assessed after 3 weeks of therapy (interim analysis day 21, n=3) before they showed any signs of distress.

**A-C.** Statistical significance as specified in Materials and Methods.

ns=not significant; ** $p \leq 0.01$; *** $p \leq 0.005$; **** $p < 0.001$
