# Supplementary material for: The PARP1 Inhibitor AZD5305 Impairs Ovarian Adenocarcinoma Progression and Visceral Metastases in Patient-derived Xenografts Alone and in Combination with Carboplatin
Source: Cancer Res Commun. 2023 Mar 27;3(3):489–500. doi: 10.1158/2767-9764.CRC-22-0423 (PMC10042207; doi:10.1158/2767-9764.CRC-22-0423)
Supplement: Supplementary Fig. S6 — Fig. S6 shows the effect of the combination with suboptimal doses of CPT or AZD5305 [file crc-22-0423-s06.pdf]

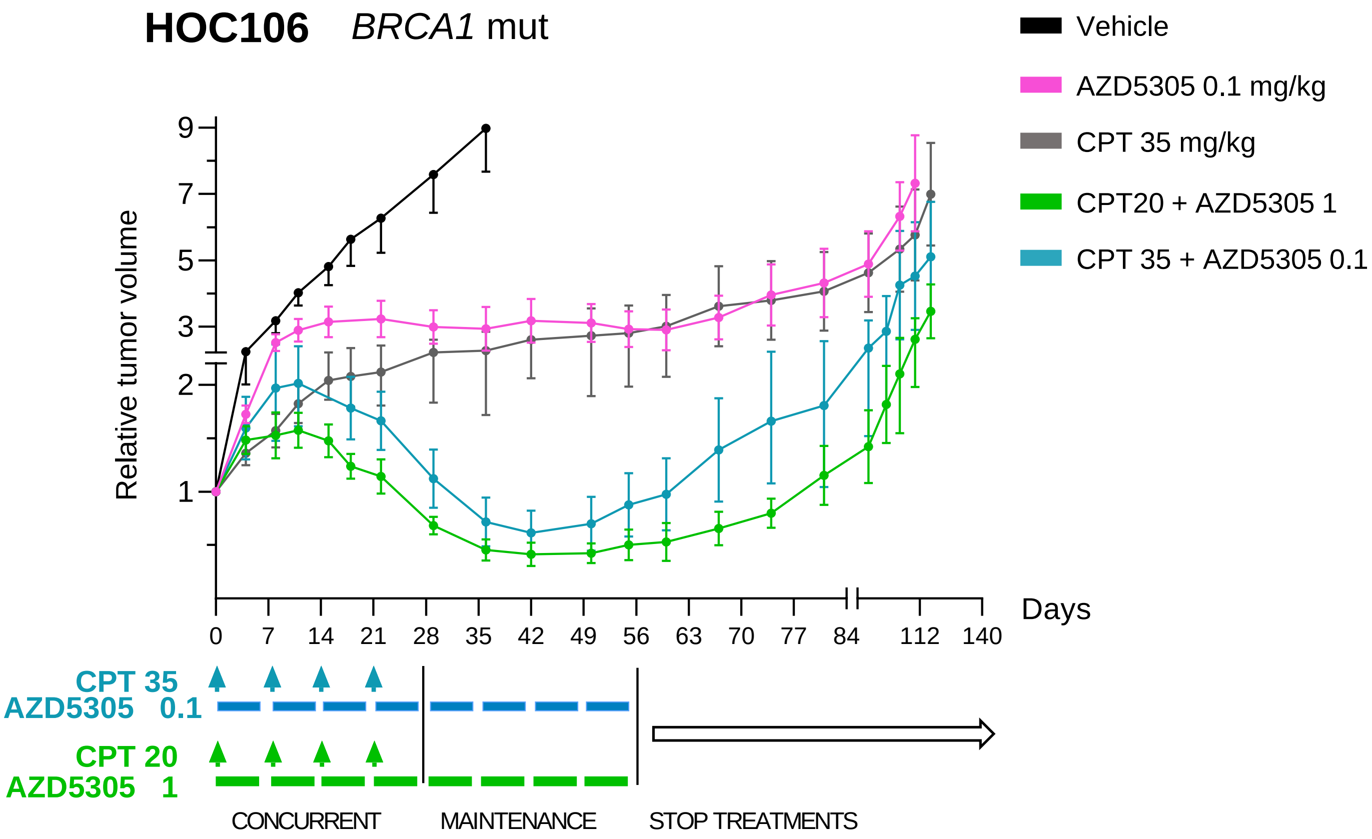

Supplementary Figure S6

Lowering the combination doses of either AZD5305 or CPT had a comparable anti-tumor efficacy.

HOC106 tumor bearing mice were given CPT (20 or 35 mg/kg), AZD5305 (0.1 or 1 mg/kg) or combination therapy (4 weeks of concurrent treatment followed by 4 weeks of AZD5305 single-agent). Data are expressed as relative tumor volume (mean ± SEM). Colored bars and arrows indicate the dosing periods. Number of mice/group=6-8.

Reducing the combination dose of either AZD5305 or CPT (i.e. CPT35 plus 0.1 mg/kg AZD5305 *vs* CPT20 plus 1 mg/kg AZD5305, *the latter is the same arm of figure 3A*) achieved a comparable antitumor effect (significantly superior to monotherapies).
