# Supplementary material for: The PARP1 Inhibitor AZD5305 Impairs Ovarian Adenocarcinoma Progression and Visceral Metastases in Patient-derived Xenografts Alone and in Combination with Carboplatin
Source: Cancer Res Commun. 2023 Mar 27;3(3):489–500. doi: 10.1158/2767-9764.CRC-22-0423 (PMC10042207; doi:10.1158/2767-9764.CRC-22-0423)
Supplement: Supplementary Fig. S7 — Fig. S7 shows the effect of the combination on OC-PDX HOC84 [file crc-22-0423-s07.pdf]

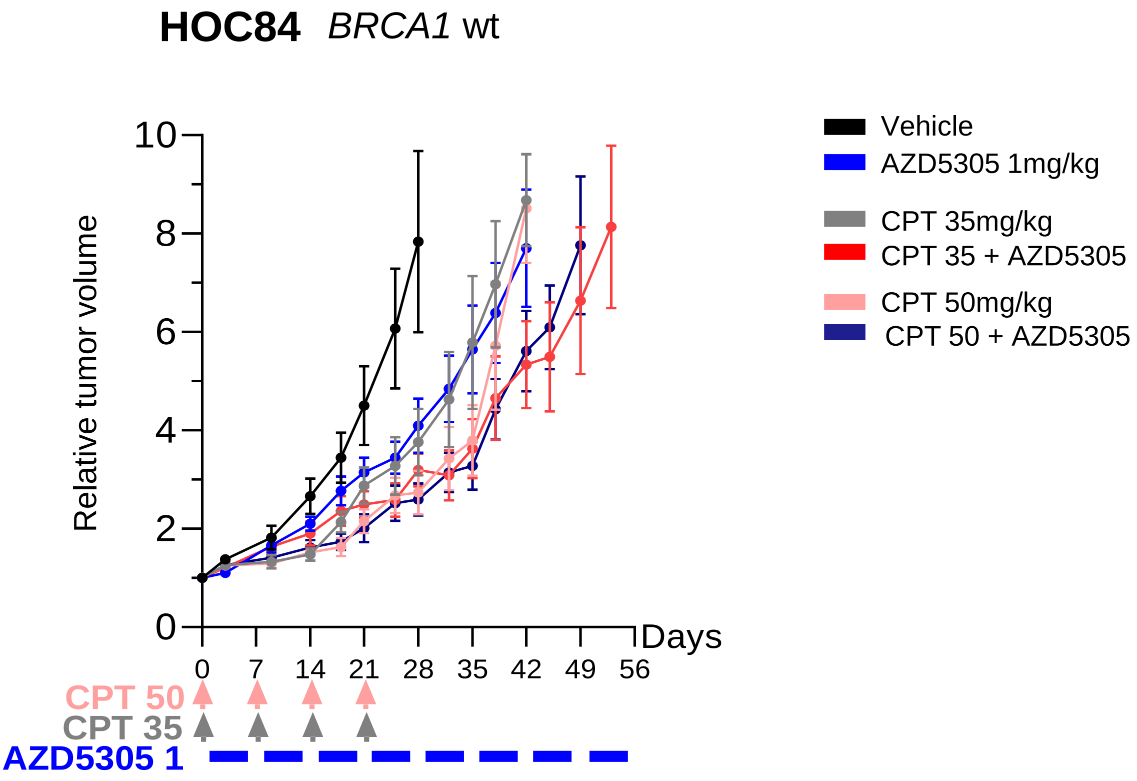

Supplementary Figure S7

AZD5305 had little effect in potentiating carboplatin (CPT) activity on OC-PDX HOC84

HOC84 tumors growing subcutaneously were randomized at a tumor volume of 170 mm^3 (SD 51.7) to be treated with CPT (35 or 50 mg/kg iv once a week, for 4 weeks) or AZD5305 (1 mg/kg orally QD, five days ON and two OFF for 8 weeks) or combination therapy (4 weeks of concurrent treatment followed by 4 weeks of AZD5305 single-agent (maintenance)). Data are expressed as relative tumor volume (mean ± SEM). Colored bars and arrows indicate the dosing periods. Number of mice/group=6-8.

The combination therapy gave no particularly benefit over the single agents.
