# Supplementary material for: The PARP1 Inhibitor AZD5305 Impairs Ovarian Adenocarcinoma Progression and Visceral Metastases in Patient-derived Xenografts Alone and in Combination with Carboplatin
Source: Cancer Res Commun. 2023 Mar 27;3(3):489–500. doi: 10.1158/2767-9764.CRC-22-0423 (PMC10042207; doi:10.1158/2767-9764.CRC-22-0423)
Supplement: Supplementary Fig. S8 — Fig. S8 shows olaparib pharmacokinetics and target engagement [file crc-22-0423-s08.pdf]

OLAPARIB PHARMACOKINETICS AND TARGET ENGAGEMENT

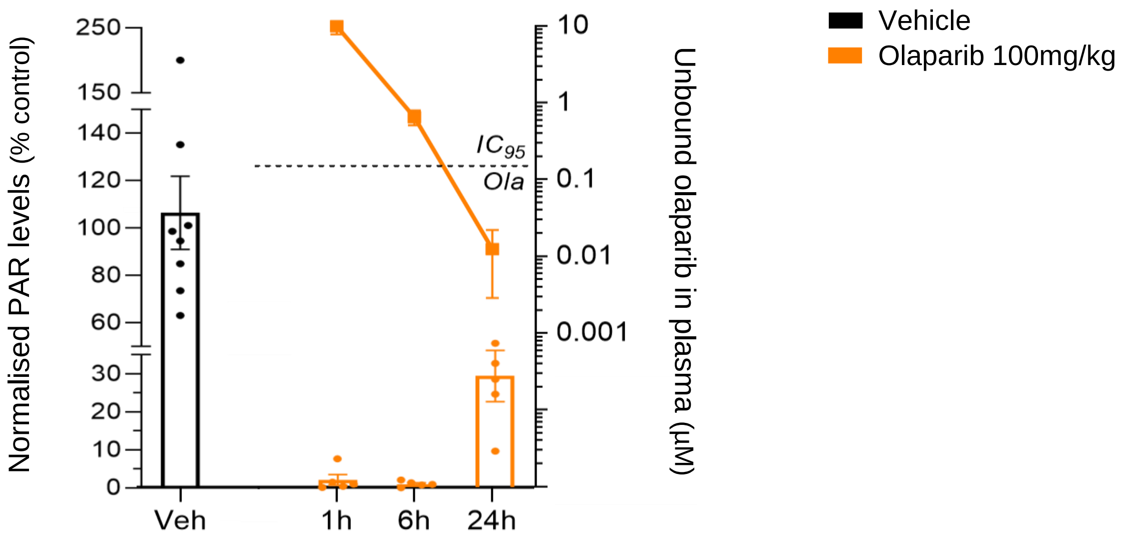

Supplementary Figure S8

Olaparib pharmacokinetics and target engagement at steady state 1, 6 and 24 h after the last of five consecutive treatments (100 mg/kg, orally QD) given to HOC107 tumor bearing mice. Unbound plasma concentration (right axis) and total PARylation in the tumor lysates (left axis). PARylation (a PD biomarker of target engagement) was more than 90% inhibited 1-6 h after dosing, but levels recovered to 30% by 24 h. Likewise after 6 h, the free plasma concentrations of olaparib dropped below the *in vitro* DLD-1 *BRCA2*-/- cells clonogenic assay IC95.
